# Constipation fonctionnelle en population générale à Cotonou (Bénin): aspects cliniques et influence des facteurs nutritionnels

**DOI:** 10.11604/pamj.2019.34.210.16945

**Published:** 2019-12-23

**Authors:** Jean Séhonou, Comlan N'déhougbea Martin Sokpon, Aboudou Raïmi Kpossou, Colette Azandjeme, Hugues Cataria, Rodolphe Koffi Vignon

**Affiliations:** 1 Clinique Universitaire d'Hépato-Gastroentérologie, Centre National Hospitalier Universitaire-Hubert Koutoukou Maga (CNHU-HKM) de Cotonou, Cotonou, Bénin; 2 Institut Régional de Santé Publique (IRSP), Ouidah, Bénin

**Keywords:** Constipation fonctionnelle, aspects cliniques, facteurs nutritionnels, Cotonou, Bénin, Afrique subsaharienne, Functional constipation, clinical aspects, nutritional factors, Cotonou, Benin, sub-Saharan Africa

## Abstract

**Introduction:**

La constipation est considérée comme rare en Afrique subsaharienne, en raison de la forte teneur en fibre de l'alimentation locale. Le but de ce travail était de décrire les différents aspects cliniques de la constipation fonctionnelle en population générale à Cotonou et de déterminer l'influence de l'alimentation dans sa survenue.

**Méthodes:**

Cette étude était menée de juillet à août 2017. La constipation était fonctionnelle si les critères de Rome IV ou de l'échelle de Bristol étaient remplis.

**Résultats:**

**A**u total, 1058 participants étaient inclus: (574 hommes, sex-ratio de 1,2; âge moyen 29 ans). La fréquence de la constipation fonctionnelle était de 24.2% (Rome IV) et de 20.4% (Echelle de Bristol). Les manifestations cliniques étaient dominées par l'émission de selles dures ou fragmentées (90,6%), les efforts de poussée lors de la défécation (78,9%), et la sensation de vidange incomplète (76,2%). Les habitudes alimentaires chez les constipés étaient: au petit déjeuner, la bouillie de maïs + beignet/arachides (39,1%), au déjeuner, la pâte de maïs (38,7%), au diner, la pâte de maïs (57,4%), et en guise de collation: la banane (35,5%). Il n'y avait pas de lien statistiquement significatif entre la constipation et respectivement le type de repas pris au petit-déjeuner (p=0,09), au déjeuner (p=0,901), en collation (p=0,09) ou au dîner (p=0,75).

**Conclusion:**

L'émission de selles dures ou fragmentées était la manifestation clinique la plus dominante chez les constipés à Cotonou. Les habitudes alimentaires n'influenceraient pas la survenue de la constipation fonctionnelle dans notre série.

## Abstract (en)

**Introduction:**

Constipation is rare in sub-Saharan Africa, due to the high intake of dietary fiber. The purpose of this work was to describe the different clinical features of functional constipation in the general population in Cotonou and to determine the impact of diet on its occurrence.

**Methods:**

This study was conducted from July to August 2017. Subjects were considered affected by functional constipation when criteria for Rome IV or Bristol stool scale were completed.

**Results:**

In total, 1058 participants were enrolled: (574 men, sex-ratio 1.2; average age 29 years). The prevalence of functional constipation was 24.2% (Rome IV) and 20.4% (Bristol stool scale). Clinical manifestations were dominated by the emission of hard or fragmented stools (90.6%), pushing efforts during defecation (78.9%), and feeling of incomplete evacuation (76.2%). Dietary habits among people affected by constipation were: at breakfast, maize meal + donut/peanuts (39.1%), lunch, corn dough (38.7%), at dinner, corn dough (57.4%), and as a snack banana (35.5%). There was no statistically significant relationship between constipation and morning meal (p=0.09), lunch meal (p=0.901), snack (p=0.09) or dinner (p=0.75) respectively.

**Conclusion:**

The emission of hard or fragmented stools was the most common clinical manifestation among people affected by constipation living in Cotonou. Dietary habits do not influence the occurrence of functional constipation in our case series.

## Introduction

La perception individuelle qu'ont les patients de même que les médecins de la constipation, influence les données épidémiologiques et cliniques de la maladie dans le monde. Néanmoins la constipation reste une maladie fréquente dans le monde. Sa prévalence varie suivant les continents: elle est de 24,2% en Amérique, 17,1% en Europe et 15,3% en Océanie [1,2]. Sa pathogénie est multifactorielle; elle fait intervenir des prédispositions génétiques les troubles de la motilité intestinale, les déséquilibres hormonaux, les effets secondaires des médicaments, des facteurs socioéconomiques [3], les troubles psychologiques [4] ou la faible consommation de fibres alimentaires et d'eau ou de boissons. Les facteurs diététiques varient d'un continent à l'autre en termes de fréquence de consommation. Au Bénin en général et à Cotonou en particulier, le régime alimentaire est basé sur les racines et tubercules (manioc, igname) et les céréales en l'occurrence le maïs (100-150kg/habitant et par an). Ces aliments de base consommés sont la plupart du temps, transformés en pâtes ou purées. Ainsi pour le maïs, la pâte de farine de maïs et l'akassa (issu de la farine de maïs fermentée) sont très consommés. Quant à l'igname, elle est surtout consommée sous forme de purée (igname pilée). Ces différentes préparations sont souvent accompagnées de sauces (contenant beaucoup d'huile). Par contre, le nombre moyen de portions de fruits et légumes consommé par personne et par jour de consommation était de 2 environ dans les deux sexes, soit en deçà des 5 portions recommandées chaque jour [5]. Ces facteurs diététiques peuvent revêtir une importance capitale pour les patients. Ces derniers veulent être soulagés de cette affection qui altère leur qualité de vie. Ils cherchent auprès du personnel soignant des conseils diététiques. Ces derniers ne reposent pas toujours sur des bases scientifiques. Au Bénin, plusieurs études ont été réalisées sur les troubles digestifs mais à notre connaissance, aucune n'a porté sur la constipation en population générale mettant l'accent sur les aspects diététiques et alimentaires. Le but de ce travail était de décrire les différents aspects cliniques de la constipation fonctionnelle en population générale à Cotonou et de déterminer l'influence de l'alimentation dans sa survenue.

## Méthodes

Il s'agissait d'une étude transversale a visée descriptive et analytique, avec un recueil prospectif des données sur une période de deux semaines allant du 28 juillet au 10 août 2017. Elle s'était déroulée dans 7 arrondissements de la commune de Cotonou, selon un échantillonnage en grappes. Etait incluse toute personne âgée d'au moins 15 ans, et résidant à Cotonou depuis au moins 6 mois. Les sujets présentant la constipation avec des signes d'alarme (hématochézie, asthénie et amaigrissement…) n'étaient pas inclus. La constipation fonctionnelle était définie selon les critères de Rome IV: début des symptômes supérieur à 6 mois et présence d'au moins 2 des symptômes suivants sur les 3 derniers mois: I) efforts de poussées (supérieur à 25% des défécations); II) selles dures ou fragmentées (supérieur à 25% des défécations); III) sensation d´évacuation incomplète (supérieur à 25% des défécations); IV) sensation de blocage anorectal (supérieur à 25% des défécations); V) manœuvres digitales (supérieures à 25% des défécations); VI) moins de 3 évacuations spontanées par semaine. La consistance des selles était évaluée grâce à l'échelle de Bristol [6], qui répartit les selles humaines en sept (07) types, sachant ce sont les selles de type 1 et 2 qui définissent la constipation. Les habitudes alimentaires étaient définies à partir des recettes alimentaires les plus fréquentes dans le sud du Bénin. Les plats proposés au petit déjeuner étaient soit: la bouillie de maïs ou de mil accompagnée de beignets d'arachides soit le thé avec du pain. Les plats proposés au déjeuner et au diner étaient composés d'un plat de résistance et de sauce.

Le plat de résistance était à base de pâte de maïs (blanche, noire, ou rouge), de riz, de pâtes alimentaires ou de farine de manioc. Quant aux sauces, elles étaient faites de tomate, gombo, arachides ou légumes. Les collations consistaient en de fruits de saison (mangue, ananas, oranges, papaye, banane, pommes), en jus de fruits ou en yaourt. Pour chaque repas, le participant avait précisé la fréquence de consommation dudit repas par semaine. La quantité de fibres alimentaires consommée par chaque participant au cours des 4 repas de la journée (petit déjeuner, déjeuner, dîner et collation), avait été calculée en additionnant les fibres contenues dans chaque constituant des repas proposés. Toutes ces préparations étaient présentées avec des photographies des plats typiques sur une feuille de régimes (à l'instar de l'échelle des selles de Bristol pour la consistance des selles). La quantité de boisson alcoolisée était mesurée comme suit: 1 consommation équivaut à un verre égale à 10 g d'alcool par unité. La collecte des données était faite lors d´une interview directe sur la base d'un questionnaire standardisé. La saisie, le traitement et l'analyse des données étaient réalisées à l'aide du logiciel SPSS 21. Les variables continues ont été exprimées en moyenne avec leur écart type. Des pourcentages ont été calculés sur les variables catégorielles. Les comparaisons ont été effectuées entre les moyennes grâce au test t de Student, entre les proportions grâce au test de Khi carré de Pearson ou du test exact de Fisher. La stratégie d'analyse multivariée par régression logistique a été utilisée pour identifier les associations significatives. Le seuil de signification statistique était de 5%. Sur le plan éthique, l'étude n'a pas été soumise au comité local d'éthique. Un consentement verbal des participants a été obtenu, et la confidentialité observée lors du recueil et du traitement des données.

## Résultats

**Caractéristiques de la population d'étude:** sur les 1058 participants inclus, il y avait 574 hommes (54,3%); sex-ratio de 1,2 (Tableau 1). L'âge moyen était de 29 ans avec des extrêmes de 15 ans et 92 ans.

**Tableau 1:** Répartition des enquêtés selon le sexe et prévalence de la constipation selon l’échelle des selles de Bristol

Sexe	Effectifs (N)	Pourcentage (%)
Masculin	574	54,3
Féminin	484	45,7
Total	1058	100
**Type de selles selon Bristol**		
Selles type 1 et 2	216	20,4
Autres types	842	79,6
Total	1058	100

**Prévalence de la constipation:** sur les 1058 participants, la constipation fonctionnelle, définie selon les critères de Rome IV, était notée chez 256 participants (24,2%), et selon l'échelle des selles de Bristol dans 216 cas (20,4%) (Tableau 1).

**Aspects cliniques:** les manifestations cliniques étaient diversifiées, mais dominées par l'émission de selles dures ou fragmentées (90,6%), et l'effort de poussée lors de la défécation (78,9%) (Tableau 2).

**Tableau 2:** Répartition des enquêtés selon les manifestations cliniques

	Constipation Rome IV			
	Oui		Non	
	N	(%)	N	(%)
**Effort de poussée**				
Oui	202	(78,9%)	302	(37,7%)
Non	54	(21,1%)	500	(62,3%)
**Selles dures ou fragmentées**				
Oui	232	(90,6%)	256	(31,9%)
Non	24	(9,4%)	546	(68,1%)
**Vidanges incomplètes**				
Oui	195	(76,2%)	219	(27,3%)
Non	61	(23,8%)	583	(72,7%)
**Manœuvres digitales**				
Oui	44	(17,2%)	31	(3,9%)
Non	212	(82,5%)	771	(96,1%)
**Impression d’obstacles**				
Oui	110	(43%)	60	(7,5%)
Non	146	(57%)	742	(92,5%)
**Moins de trois selles par semaine**				
Oui	142	(55,5%)	62	(7,7%)
Non	100	(39,1%)	316	(39,4%)

**Signes associés à la constipation:** la constipation était le plus souvent associée aux douleurs abdominales (30,7%), aux ballonnements abdominaux (24,4%) et à l'anorexie (23,8%) des cas (Tableau 3).

**Tableau 3:** Répartition en fonction des signes associés à la constipation

	Effectifs (N)	Pourcentage (%)
Ballonnement abdominal	258	24,4
Douleurs abdominales	325	30,7
Diarrhée	169	16
Emission de sang par l’anus	57	5,4
Asthénie intense	237	22,4
Amaigrissement	240	22,7
Anorexie	252	23,8
**Total**	**1058**	**100**

**Facteurs alimentaires:** les habitudes alimentaires les plus représentées chez les constipés étaient: au petit déjeuner la bouillie de maïs + beignet/arachides (39,1%); au déjeuner, la pâte de maïs (38,7%); au diner, la pâte de maïs (57,4%); et en collation, la banane (35,5%). De plus, 60,2% (154 sur 256) de constipés avaient un régime riche en fibres alimentaires. Cependant, il n'y avait pas de lien statistiquement significatif entre la constipation et respectivement le type de repas pris au petit-déjeuner (p=0,09), au déjeuner (p=0,901), en collation (p=0,09) ou au dîner (p=0,75). De plus, il n'était pas trouvé de lien entre le régime riche en fibres et la constipation (p=0.827) (Tableau 4). Par contre, il existait un lien entre l'hydratation et la constipation (p=0,024). En effet, consommer au moins 2500 ml d´eau par jour protègerait contre la survenue de la constipation.

**Tableau 4:** Répartition des enquêtés selon leur régime journalier en fibre alimentaire

	Constipation fonctionnelle selon les critères de Rome IV				Analyse bilatérale exacte		
	Oui		Non		OR	IC (95%)	p-value
	**N**	**(%)**	**N**	**(%)**			
Régime riche en fibres	154	(60,2%)	475	(59,2%)	-	-	0,827
Régime pauvre en fibres	102	(39,8%)	327	(40,8%)	-	-	
**Total**	**256**		**802**				

## Discussion

La prévalence de la constipation varie en fonction de la méthode diagnostique. La prévalence de la constipation fonctionnelle à Cotonou était de 24,2%, en se basant sur les critères de Rome IV. Cette prévalence se rapproche de celle trouvée en Italie par Cottone C *et al*. [7] dans une étude prospective réalisée en 2014 qui était de 24% (Critères de ROME II). Higgins PD et Johanson JF [8] avaient également trouvé des résultats proches des nôtres en Amérique (27,2%). D'autres auteurs avaient trouvé par contre des prévalences plus basses. Pare *et al*. [9] au Canada suite à une enquête par questionnaire envoyée aux participants avaient trouvé une prévalence de 16,7% (critères de ROME I). En se basant sur l'échelle des selles de Bristol, la prévalence de la constipation fonctionnelle dans notre série était de 20,4%. Cette prévalence est proche de celle retrouvée par Cottone C [7] *et al*. en Italie (27,5%). Par contre, Ray G *et al*. [10] en Inde avaient trouvé une prévalence plus élevée (67,9%), au décours d'une enquête par questionnaire auto administré. Cette variabilité de la prévalence de la constipation pourrait s´expliquer non seulement par des méthodes diagnostiques différentes mais aussi par les techniques de collecte utilisées. Quel que soit la méthode utilisée, la fréquence de la constipation était élevée à Cotonou. Cette étude ne conforte pas les affirmations antérieurement avancées. Les signes cliniques de constipation dans notre série sont variés, dominés par les selles dures et fragmentées. Basotti G *et al*. [2] ont plutôt trouvé en Italie en 2004, 5% de sujets ayant eu moins de 3 selles par semaine, 11,7% de sujets ayant eu recours à des manœuvres digitales et 10,7% de sujets ayant une vidange incomplète.

Cette variabilité pourrait s'expliquer par le fait que les symptômes sont des signes subjectifs, dont la perception varie d´un individu à un autre. Les habitudes alimentaires les plus représentées chez les personnes constipées étaient: au petit déjeuner, la bouillie de maïs + beignet/arachides (39,1%); au déjeuner, la pâte de maïs (38,7%), au diner, la pâte de maïs (57,4%), et en collation, la banane (35,5%). Chez les personnes non constipées, le maïs restait l'aliment le plus consommé avec: au petit déjeuner la bouillie de maïs + beignet/arachides (32,7%), au déjeuner la pâte de maïs (35,9%), au diner, la pâte de maïs (59%) et en collation, la banane (41,4%). Ces résultats sont similaires à ceux rapportés par Mitchikpe E *et al*. [11] suite à une enquête menée dans 6 villes du Bénin. Ils avaient remarqué que le maïs (sous diverses formes) était l'aliment le plus consommé à Cotonou au petit déjeuner, au déjeuner et au diner. L'élément le plus souvent cité dans l'alimentation comme étant un facteur de risque de la constipation reste le régime pauvre en fibres alimentaires. Dans notre étude, nous n'avons pas trouvé de lien entre le régime en fibres alimentaires et la survenue de la constipation. Nos résultats sont proches de ceux déjà décrits dans la littérature [12-15]. Au-delà de la composition en fibres de la ration alimentaire, il faudrait tenir compte de la qualité des dites fibres (solubles ou insolubles), du mode de cuisson, et de la nature des condiments qui accompagnent les fibres alimentaires. Au Bénin les plats de résistance sont souvent accompagnés de sauces particulièrement riches en graisse. Plus la sauce est grasse et contient des protéines animales, plus le repas est socialement valorisé.

Les graisses sont connues pour ralentir le transit colique (et donc favoriser la constipation) [16]. Il y avait un lien statistiquement significatif entre la consommation d´eau et la constipation (p=0,024). La consommation d'environ 2500ml d'eau par jour aurait un rôle protecteur contre la constipation. Nos résultats sont proches de ceux publiés par d'autres auteurs. Borre M [17] faisait remarquer que la consommation quotidienne d'au moins 2L d'eau améliorait les symptômes de la constipation. Ainsi donc la constipation est bien présente à une fréquence relativement élevée dans cette ville d'Afrique subsaharienne; de plus, la richesse traditionnelle en fibres de l'alimentation locale ne semble pas protéger totalement la population. Cela rejoint les données antérieurement décrites par Camara *et al*. [18]. Il était jusqu'à un passé récent considéré que le temps de transit colique variait en fonction du continent. Ainsi l'occidental aurait un transit moyen de 70 heures avec 100 g de selles; l'africain moyen aurait un transit de 36 heures en moyenne avec un poids de selles de 450 g en raison de son régime riche en végétaux [19]. Notre étude infirme ces notions. Ces données nous incitent à la prudence pour ce qui concerne les recommandations diététiques aux patients porteurs de constipation en Afrique subsaharienne. Certains patients abusent de fruits et légumes avec le risque de désorganiser leur régime alimentaire. L´attitude la meilleure pourrait être de faire aux patients une auto observation du régime alimentaire sur plusieurs semaines. Au décours de cette période le soignant et le patient seront face à deux cas de figure: dans le premier cas, la constipation est manifestement liée à la prise de certains aliments. Un régime de restriction et non d'éviction desdits aliments sera prônée. Une évaluation se fera dans les mois qui suivent. Dans le deuxième cas, quel que soit le régime alimentaire, la constipation persiste. L'accent pourrait être mis sur les autres recommandations non diététiques (massages, traitements pharmacologiques, lutte contre les troubles psychiatriques: anxiété et dépression en particulier).

## Conclusion

Les signes cliniques de constipation dans notre série sont dominés par les selles dures et fragmentées. L'hydratation insuffisante était le facteur associé à la constipation dans notre série. Par contre, la teneur en fibre des aliments n'influencerait pas la survenue de la constipation fonctionnelle dans notre série. Ce travail confirme la complexité de l'étiopathogénie de la constipation fonctionnelle. Il invite à en avoir une vision holistique qui débouchera sur un traitement personnalisé, individualisé si possible.

### Etat des connaissances actuelles sur le sujet

- La constipation est considérée comme rare en Afrique subsaharienne, en raison de la forte teneur en fibre de l'alimentation locale;
- La constipation serait favorisée par un régime alimentaire pauvre en fibre, le manque d'hydratation suffisante, le manque d'activité physique régulière;
- La stratégie thérapeutique peut faire intervenir des règles hygiéno-diététiques, visant à conseiller une alimentation riche en fibres solubles, et la consommation suffisante d'eau riche en minéraux surtout le magnésium.

### Contribution de notre étude à la connaissance

- La prévalence de la constipation fonctionnelle à Cotonou était de 24,2%, en se basant sur les critères de Rome IV;
- Les signes cliniques de constipation dans notre série sont variés, dominés par les selles dures et fragmentées;
- La majorité des constipés avaient un régime riche en fibres alimentaires (60,2%), cependant, il n'y avait pas de lien statistiquement significatif entre la constipation le régime riche en fibres et la constipation (p=0.827). Au Bénin, les plats de résistance sont souvent accompagnés de sauce particulièrement riches en graisse, or la graisse ralenti le transit colique et donc pourrait favoriser la constipation.

## Conflits d’intérêts

Les auteurs ne déclarent aucun conflit d´intérêts.
